# Socioeconomic differences in the reduction of face-to-face contacts in the first wave of the COVID-19 pandemic in Germany

**DOI:** 10.1186/s12889-022-14811-4

**Published:** 2022-12-23

**Authors:** Julia Waldhauer, Florian Beese, Benjamin Wachtler, Sebastian Haller, Carmen Koschollek, Timo-Kolja Pförtner, Jens Hoebel

**Affiliations:** 1 Department of Epidemiology and Health Monitoring, Division of Social Determinants of Health, Robert Koch Institute, Berlin, Germany; 2 Department of Infectious Disease Epidemiology, Healthcare-Associated Infections, Surveillance of Antibiotic Resistance and Consumption, Robert Koch Institute, Berlin, Germany; 3 Research Methods Division, Faculty of Human Sciences, University of Cologne, Cologne, Germany

**Keywords:** COVID-19, SARS-CoV-2, Educational status, Occupational status, Contact reduction, Social epidemiology

## Abstract

**Background:**

The COVID-19 pandemic has led to physical distancing measures to control the spread of SARS-CoV-2. Evidence on contact dynamics in different socioeconomic groups is still sparse. This study aimed to investigate the association of socioeconomic status with private and professional contact reductions in the first COVID-19 wave in Germany.

**Methods:**

Data from two especially affected municipalities were derived from the population-based cross-sectional seroepidemiological CORONA-MONITORING lokal study (data collection May–July 2020). The study sample (*n* = 3,637) was restricted to working age (18–67 years). We calculated the association of educational and occupational status (low, medium, high) with self-reported private and professional contact reductions with respect to former contact levels in the first wave of the pandemic. Multivariate Poisson regressions were performed to estimate prevalence ratios (PR) adjusted for municipality, age, gender, country of birth, household size, contact levels before physical distancing measures, own infection status, contact to SARS-CoV-2 infected people and working remotely.

**Results:**

The analyses showed significant differences in the initial level of private and professional contacts by educational and occupational status. Less private contact reductions with lower educational status (PR low vs. high = 0,79 [CI = 0.68–0.91], *p* = 0.002; PR medium vs. high = 0,93 [CI = 0.89–0.97], *p* = 0.001) and less professional contact reductions with lower educational status (PR low vs. high = 0,87 [CI = 0.70–1.07], *p* = 0.179; PR medium vs. high = 0,89 [CI = 0.83–0.95], *p* = 0.001) and lower occupational status (PR low vs. high = 0,62 [CI = 0.55–0.71], *p* < 0.001; PR medium vs. high = 0,82 [CI = 0.77–0.88], *p* < 0.001) were observed.

**Conclusions:**

Our results indicate disadvantages for groups with lower socioeconomic status in private and professional contact reductions in the first wave of the pandemic. This may be associated with the higher risk of infection among individuals in lower socioeconomic groups. Preventive measures that a) adequately explain the importance of contact restrictions with respect to varying living and working conditions and b) facilitate the implementation of these reductions especially in the occupational setting seem necessary to better protect structurally disadvantaged groups during epidemics.

**Supplementary Information:**

The online version contains supplementary material available at 10.1186/s12889-022-14811-4.

## Introduction

With ongoing research on the COVID-19 pandemic [1] and its implications, more and more studies found socioeconomic inequalities in different COVID-19 outcomes. A systematic scoping review of international studies on socioeconomic inequalities in COVID-19 concluded that outcomes, such as incidence, hospitalizations, or mortality related to COVID-19, were higher in socioeconomically disadvantaged groups [2]. Those findings were confirmed by more recent studies from later phases of the pandemic [3]. Tentative first explanations of these inequalities included higher proportions of comorbidities and certain environmental conditions like the ongoing active employment in essential occupations, the usage of public transport, the lacking ability to work from home, or crowded living conditions [4, 5] in disadvantaged populations lead to higher infection rates and worse COVID-19 trajectories, respectively [6, 7]. Especially at the beginning of the global pandemic, when vaccinations were not yet available, non-pharmaceutical interventions (NPI) appeared to be effective in reducing the COVID-19 transmissions [8] and hence in lowering the reproduction number in several countries [8, 9]. The legal provisions on mitigation strategies had an immense impact on peoples’ everyday life and societies worldwide. NPIs resulted in abrupt changes in the educational, professional and business life worldwide [10] and affected people differently depending on their socioeconomic background [11, 12].

A key strategy for preventing infections is to limit close contacts. On an international level, Liu et al. (2021) found that the majority of studies considering individual contact data were conducted in European countries and analyzed contact patterns during the mitigation period in spring 2020. Here, most of the studies reported 2 to 5 contacts per day compared to 7 to 26 contacts per day in the pre-pandemic setting on average [13]. The COVID-19 Health Behavior Survey (CHBS) showed that household size and paid work as well as being male contributed to a positive association with contact numbers during March and April 2020 in all countries [14]. However, most of the current knowledge on socioeconomic inequalities in mobility reductions and the compliance with physical distancing measures comes from ecological studies. An ecological analysis of county-level socioeconomic data in the United States during the first lockdown period concluded that counties with higher proportions of people with lower educational status were associated with a lower stay-at-home behavior. Higher proportions of unemployment were by contrast associated with higher stay-at-home behavior [15]. Another study with a similar approach using neighborhood income indicated that the adherence to governmental orders on physical distancing was lower in lower-income neighborhoods compared to higher-income neighborhoods [16]. This is also reflected by an ecological study from England considering area-level occupational exposures on mobility reductions in the lockdown period in spring 2020. Areas with a higher proportion of lower-paid and lower-educated workers or workers in precarious jobs showed lower mobility reductions compared to areas of higher average socioeconomic position [17].

While ecological analyses provide a quick and resource-preserving approximation of regional effectivity of mitigation strategies and give advice of certain clustering and high-risk regions during the pandemic, corresponding results cannot infer implications on an individual level [18]. To address individual risks, needs and barriers along with mitigation strategies, individual contact data is needed to provide comprehensive information that might support targeted interventions and strategies. Furthermore, a study from Germany concluded that individual contact data reflected infection dynamics better than aggregated mobility data [19]. However, studies that analyzed individual data on preventive behaviors, especially on contact reductions in Germany with respect to socioeconomic stratifications, are still rare [20, 21]. Lüdecke and von dem Knesebeck (2020) reported an educational gradient in the reduction of “personal meetings and contacts” and in the adaption of “school or work situation” with the odds of both outcomes were lowest in low-educated individuals compared to high-educated individuals but lack information on occupational status and differences regarding professional contacts. Results from the United States indicate only for women increased odds of staying at home or performing physical distancing in mid-income groups compared to low- and high-income groups, and among women with trade or vocational education compared to women with high-school education or less [22].

There is a lack of studies with an exclusive focus on socioeconomic inequalities in contact reductions, especially those focusing occupational status and comparing different settings of personal contacts. Therefore, the objective of this paper was to analyze educational and occupational differences in the change of private and professional contacts on an individual level before and after 18 March 2020, when the former German government introduced the first nation-wide contact reductions and physical distancing measures to prevent an overwhelming of the German health system [23].

## Methods

### Data

We analyzed data of the CORONA-MONITORING lokal study (CoMoLo), initiated in 2020 by the Robert Koch Institute, the German national public health institute, to estimate the spread of SARS-CoV-2 infections using a population-based, seroepidemiological approach. Cross-sectional investigations were conducted in four German municipalities that were especially affected during the first wave of the pandemic with a cumulative SARS-CoV-2 incidence of over 500 cases per 100,000 inhabitants one month before the start of data collection. A proportional random population registry-based sample of adults 18 years and older was invited to study centers in the corresponding municipalities for participating on site or on request for being visited at home by the study team. Two weeks after participation in blood and swab sampling, a detailed questionnaire survey was conducted either web-based or by telephone. The questionnaire included information about demographics, COVID-19 diagnosis and symptoms, health care, health behavior, social relations, and private and professional contacts [24].

We analyzed data of two out of the four municipalities investigated in the CoMoLo study, which were Kupferzell and Bad Feilnbach in southern Germany. We selected these municipalities because data collection periods were comparatively close to the introduction of the first physical distancing measures in Germany (18 March 2020) and, therefore, had short recall periods for retrospective questions on contact reductions during the first wave of the pandemic. In Kupferzell, data collection was performed between 20 May and 9 June 2020 with a response rate of 63% [25]. In Bad Feilnbach, data collection was performed from 23 June to 4 July 2020 with a response quote of 55% [26]. In terms of population and area characteristics, both municipalities are comparable. Both are located in rural south-western Germany, had similar outbreak trajectories in the beginning of the first wave of the pandemic and show comparable demographics in terms of age and socioeconomic structure [27].

### Variables

#### Private and professional contact reductions

We computed two binary outcome variables concerning private and professional contact reductions after 18 March 2020. Contacts were defined in the study as “direct face-to-face interactions taking longer than 15 min and with an interpersonal distance less than 1.5 m”. Participants were asked: “Did the number of private contacts such as relatives, friends, or neighbors (question one)/of professional contacts with colleagues or employees at work (question two) change after 18 March 2020?”. Possible answers were “No change”, “Yes, less contacts”, and “Yes, more contacts”. Private or professional contact reduction was given when individuals ticked “Yes, less contacts”. No contact reduction was given when individuals ticked “Yes, more contacts” or “No change”.

#### Socioeconomic variables

Educational status was measured according to the International Standard Classification of Education from 2011 (ISCED-11) [28]. Education was categorized into the levels “low”, “medium”, and “high”. “Low” refers to at most secondary school education without vocational education. “Medium” refers to at least having a general qualification for university entrance or completed a vocational training, while “high” refers to having completed a tertiary education.

Occupational status was measured according to the International Socio-Economic Index of Occupational Status (ISEI-08) [29]. We computed quintiles of the metric ISEI-08 scale and built three categories for our analyses, i.e., “low (Q1)”, “medium (Q2-4)”, and “high (Q5)”. Additionally, we defined a fourth category referring to individuals not (or no longer) employed or not specified, or to participants who are not part of the regular labor market (e.g. students, training course participants, people in parental leave). This group is retained for the calculations to avoid missing values, but is not interpreted in terms of content due to the diversity within the group and because of too small case numbers for single examinations.

#### Covariates

In the multivariate analyses, we used several covariates: municipality, gender, age, household size and country of birth (Germany vs. foreign-born: born in another country than Germany). Assuming that individuals who have more social contacts are more able to reduce contacts thereafter, we controlled for the former contact level in the analyses. Participants were asked to retrospectively report their weekly private contacts before physical distancing measures (18 March 2020): “In an average week before 18 March 2020, how many direct contacts to relatives, friends, and neighbors (question one)/ how many direct contacts to colleagues (question two) did you have at least once per week that were not members of your household?”. Possible answers were “No regular contact”, “1–5 contacts/week”, “6–10 contacts/week”, and “ > 10 contacts/week”.

Given the higher probability of lower professional contacts when working from home, we also included working remotely as a covariate in the models with occupational status as a predictor for professional contact reduction. To the question “What applied to your professional situation in the period after 18 March 2020?” one out of further answers was: “I did most of my work at home (e.g., working remotely).” Additionally, we included if participants have ever been infected by SARS-CoV-2. We defined an infection if either the participant was tested seropositive for SARS-CoV-2 IgG antibodies (Euroimmun SARS-CoV-2-S1 IgG-ELISA: ratio ≥ 1.1), had a positive SARS-CoV-2-RT-PCR swab test during the study or self-reported a positive PCR test prior to the study. We furthermore included a variable indicating whether individuals had contact to infected people, assuming that those individuals were more aware to risk of infection and might reduce their contacts as they have been exposed to SARS-CoV-2, as well.

### Statistical analyses

Analyses were restricted to the sample aged between 18 and 67 years as this refers to the working age in Germany. Individuals who were not employed or were not part of the regular labor market were retained in the multivariate analyses but were not reported or interpreted due to the heterogeneity within this group. Descriptive relative frequencies and multivariate analyses were calculated with weighted data, which adjusted the net samples to match the official populations statistics according to age, gender, and education in the respective municipalities [24]. Statistical analyses were performed for both municipalities together and separately. Because of the high outcome prevalence in the cross-sectional data and the retrospective design of the questions on contact reductions, we used Poisson regression to estimate prevalence ratios (PR) [30].

We performed multivariate analyses with different sets of covariates. The basic set of covariates includes municipality, age, gender, country of birth, household size, level of contacts before 18 March 2020 for both private and professional contacts, own infection status and contact to SARS-CoV-2 infected people. In a second model we adjusted for education and occupational status respectively. In the models for professional contact reductions we included working remotely additionally. We used Stata statistical software version 16.1 for statistical analyses [31]. Descriptive figures and Pearson’s χ^2^-tests were computed using R statistical software version 4.1.2 [32].

The sample of the analyses is described in Table. 1.

**Table 1:** Description of the study sample by municipality (*n* = 3,635)

	Kupferzell *n* = 1900 (%)		Bad Feilnbach *n* = 1735 (%)
**Gender**			
Female	980 (47.4)		938 (49.6)
Male	920 (52.6)		797 (50.4)
Missing	0 (-)		0 (-)
**Age group**			
18–24	229 (9.6)		186 (10.5)
25–44	851 (40.5)		667 (38.2)
45–59	566 (35.0)		615 (36.2)
60–67	254 (14.9)		267 (15.1)
Missing	0 (-)		0 (-)
**Country of birth**			
Germany	1532 (91.2)	1484 (95.5)	
Foreign-born	145 (8.8)	76 (4.5)	
Missing	223 (-)	175 (-)	
**Household size**			
One person	156 (8.7)	172 (10.2)	
More than one person	1713 (91.3)	1548 (89.8)	
Missing	31 (-)	15 (-)	
**Own SARS-CoV-2 infection, lifetime**			
No	1551 (91,4)	1487 (93,6)	
Yes	148 (8,6)	99 (6,4)	
Missing	201 (-)	149 (-)	
**Contact to SARS-CoV-2 infected people**			
No	1164 (69,2)	1153 (73,6)	
Yes	527 (30,8)	418 (26,4)	
Missing	207 (-)	164 (-)	
**Working remotely**			
No	1401 (79.1)	1320 (78.6)	
Yes	431 (20.9)	384 (21.4)	
Missing	68 (-)	31 (-)	
**Education**			
Low	270 (9.3)		224 (8.6)
Medium	980 (56.3)		874 (54.5)
High	639 (34.4)		632 (36.9)
Missing	11 (-)		6 (-)
**Occupatoinal Status**			
Low (Qintile 1)	251 (18.8)		251 (19.7)
Medium (Quintile 2—4)	786 (46.9)		658 (41.1)
High (Quintile 5)	280 (14.9)		282 (16.6)
Not employed/not specified	342 (19.4)		359 (22.6)
Missing	241 (-)		185 (-)
**Private weekly contact level before 18 March 2020**			
No regular contacts	206 (13.9)		185 (12.0)
1 to 5	717 (42.2)		702 (45.4)
6 to 10	466 (26.6)		415 (25.4)
More than 11	302 (17.3)		266 (17.1)
Missing	209 (-)		167 (-)
**Professional weekly contact level before 18 March 2020**			
No regular contacts	180 (11.1)		191 (12.8)
1 to 5	439 (26.6)		464 (30.2)
6 to 10	361 (21.8)		290 (18.1)
More than 11	493 (28.1)		395 (24.9)
Not applicable	215 (12.5)		226 (14.0)
Missing	211 (-)		169 (-)
**Private contacts after 18 March 2020**			
Reduction	1362 (83.5)		1183 (78.9)
No reduction	243 (16.5)		301 (21.1)
Missing	295 (-)		251 (-)
**Professional contacts after 18 March 2020**			
Reduction	1004 (70.5)		770 (62.4)
No reduction	367 (29.5)		437 (37.6)
Missing	529 (-)		528 (-)

n = crude number of observations, (%) = weighted percentage according to data of the official population statistics regarding age, gender and education; (%) do not necessarily add up tp 100% due to rounded values

## Results

### Private contacts and reduction by educational status

**Fig. 1:**
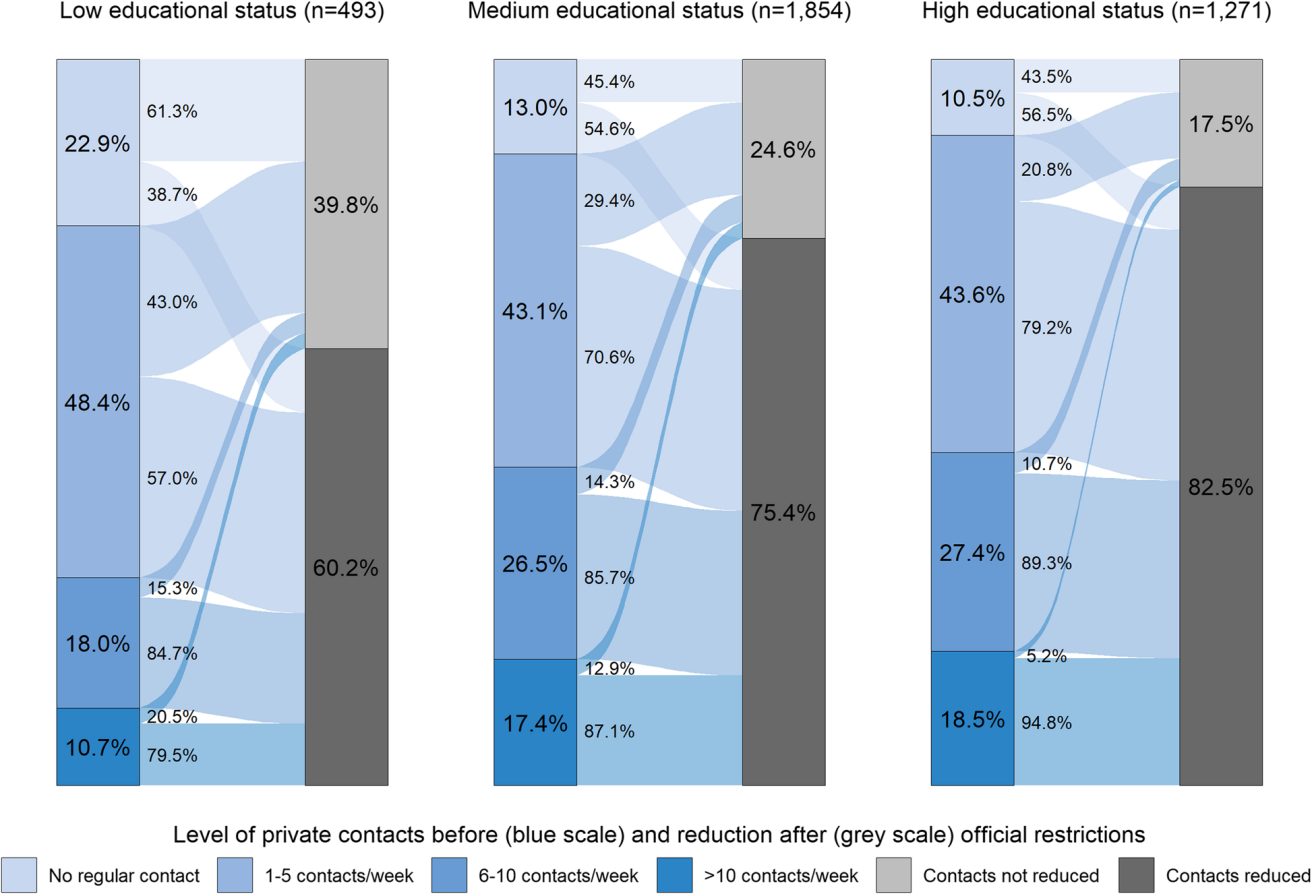
Private contact levels before and private contact reduction after 18 March 2020 by educational status. n unweighted; % weighted according to data of the official population statistics regarding age, gender and education; Pearson’s χ^2^-test with Rao & Scott adjustment between education and private contact reduction: *p* < 0.001

The comparison by educational status showed a social gradient both in the number of private contacts and their reduction after 18 March 2020. With rising education, people reported higher initial contact levels. People with high education reduced their private contacts to a larger extend (82.5%) compared to people with medium (75.4%) or low educational status (60.2%) (see Fig. 1). The respective share of private contact reductions between the former contact levels showed congruent gradients over all educational groups, comparable to the main results. For example, in the group with more than ten contacts per week (in Fig. 1 last box in the left blue scale), 79.5% of the low education group, 87.1% of the medium education group and 94.8% of the high education group reduced their contacts.

Regardless of socioeconomic determinants, women reported fewer private contacts compared to men and reduced them to a slightly larger extend after 18 March 2020 (see Fig. App. 1 in Additional File 1).

### Professional contacts and reduction by educational and occupational status

In comparison to private contacts, the participants reported overall higher initial rates of professional contacts and fewer contact reductions after 18 March 2020.

In the total sample, people with high educational status showed a larger number of professional contacts before and reported a reduction after 18 March 2020 more frequently (71.5%) compared to people with medium (55.4%) or low educational status (47.6%).

**Fig. 2:**
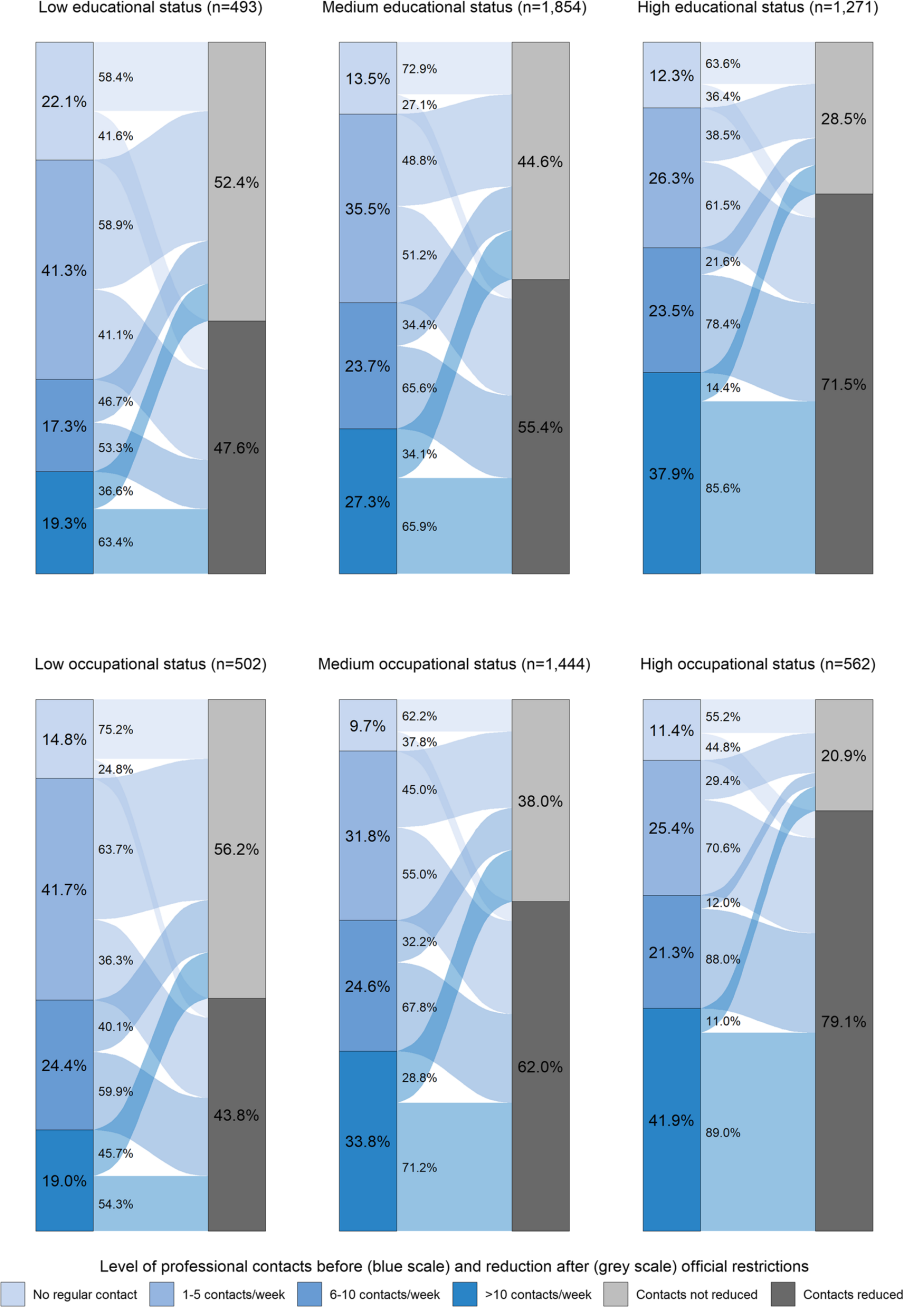
Professional contact levels before and professional contact reduction after 18 March 2020 by educational and occupational status. n unweighted; % weighted according to data of the official population statistics regarding age, gender and education; Pearson’s χ^2^-test with Rao & Scott adjustment between education/occupation and professional contact reduction: Educational status: *p* < 0.001; Occupational status: *p* < 0.001

The respective share of professional contact reductions in the former contact levels showed congruent gradients over all educational groups, comparable to the main results for private contact reduction by educational status. In the group with no regular contacts per week (in Fig. 2 first box in the left blue scale), those people with low education showed higher proportions of professional contact reduction (41.6%) than people with medium (27.1%) or high education (36.4%).

The stratification by occupational status showed a higher initial contact level compared to the results by educational status and an even steeper gradient in the reduction of professional contacts after 18 March 2020. In the total sample, 43.8% of the low occupation group, 61.9% of the medium occupation group and 79.9% of the high occupation group reduced their professional contacts (see Fig. 2). Also, the respective share of professional contact reductions in the former contact levels showed congruent gradients over all occupational status groups.

The results for professional contacts without socioeconomic stratification showed some differences between female and male, with higher rates of former professional contact levels in men and rather no difference by gender in the percentages of reduction after 18 March 2020 (see Fig. App. 1 Additional File 1).

### Prevalence ratios of private and professional contact reduction by educational and occupational status

Taking all covariates (municipality, age, gender, country of birth, household size, contact level before 18 March 2020, contact to SARS-CoV-2 infected people and own infection status) into account, educational status was positively associated with private contact reductions after 18 March 2020. Compared to the group with high educational status, people with low educational status showed a 21% higher prevalence of not reducing their private contacts, those with medium educational status 7% (see Table. 2: M1a). Differences in private contact reduction by educational status remained significant after adjusting for occupational status (see Table 2. M2a).

**Table 2:** Reduction of private and professional contacts after 18 March 2020 by educational and occupational status (*n* = 2,656)

**Reduction of private contacts**	**Educational status** (ref. high)		**Occupational status** (ref. high)	
	Low	Medium	Low	Medium
M1a: Confounder model^*^	0.79 (0.68;0.91) ***p* = 0.002**	0.93 (0.89;0.97) ***p* = 0.001**		
M2a: M1a + adjusting for occupational status	0.81 (0.70;0.94) ***p* = 0.006**	0.95 (0.91;0.99) ***p* = 0.028**		
**Reduction of professional contacts**				
M1b: Confounder model^*^	0.76 (0.62;0.93) ***p* = 0.008**	0.80 (0.75;0.85) ***p* < 0.001**	0.58 (0.52;0.66) ***p* < 0.001**	0.78 (0.74;0.83) ***p* < 0.001**
M2b: M1b + adjusting for occupational resp. educational status	0.87 (0.70;1.07) *p* = 0.179	0.89 (0.83;0.95) ***p* = 0.001**	0.62 (0.55;0.71) ***p* < 0.001**	0.82 (0.77;0.88) ***p* < 0.001**
M3b: M2b + adjusting for working remotely	0.93 (0.76;1.15) *p* = 0.500	0.92 (0.86;0.98) ***p* = 0.011**	0.69 (0.61;0.78) ***p* < 0.001**	0.89 (0.83;0.94) ***p* < 0.001**

^*^Prevalence ratios with 95% confidence intervals (in brackets) adjusted for municipality, age, gender, country of birth, household size, contact level before 18 March 2020, contact to SARS-CoV-2 infected people, own infection (lifetime); Significant p-values (*p* < 0.05) in bold

Similarly, we observed educational differences in professional contact reductions. When adjusting for occupational status and working remotely, differences in contact reductions by educational status only remained statistically significant between medium and high educational status (see Tab. 2 M2b and M3b).

The models for professional contact reduction by occupational status showed similar gradual associations of less contact reductions in low and medium occupational status groups on a more pronounced level (see Tab. 2 M1b-M3b). The prevalence of not reducing professional contacts after 18 March 2020 was elevated about more than 30% in the low occupational group and more than 10% in the medium occupational group compared to people with high occupational status and adjusting for educational status and working remotely (see Table. 2 right side M2b and M3b). The separate analyses for each municipality do not reveal any major discrepancies compared to the joint analyses (see Tab. App. 1 and Tab. App. 2 in Additional File 1).

## Discussion

In this study, we analyzed educational and occupational differences in private and professional contact reductions using data from the CoMoLo study focusing on two especially affected municipalities in the first wave of the COVID-19 pandemic in Germany. We found higher initial contact levels and a higher prevalence of contact reductions with rising educational and occupational status. In general, people reduced more private contacts compared to professional contacts. The prevalence of not reducing professional contacts was stronger associated with occupational status compared to educational status and was highest for individuals with low occupational status. Our study helps to better understand the context in which socioeconomic differences in infection rates developed during the first pandemic wave in Germany. One major pathway to socioeconomic differences in SARS-CoV-2 infections is the unequal exposure to the virus [33] and the level of exposure depends on the number and circumstances of private and professional contacts.

There is international evidence that the risk of infection in the early phases of the pandemic was higher in socioeconomically better-off groups, e.g. due to former higher mobility rates (business travel, vacation travel) [34], which is compatible with our findings of higher private and professional contacts in higher educational and occupational groups before implementation of the mitigation strategies [35]. However, as the pandemic progressed, the dynamics reversed and the risk of infection was higher in socioeconomically disadvantaged groups [36–38], which also can be seen as a result of less contact reductions in lower educational and occupational groups, as found in our analysis.

The results are consistent with existing preliminary work in terms of the overall tendency to reduce one's contacts during the first pandemic period [13]. The results further illustrate that this applies to both private and professional contacts. But even in places where the incidence was particularly high, as in the municipalities studied here, socioeconomic differences in preventive behavior was observable and was not leveled out by the epidemic situation. Other international studies have already shown higher contact rates among men in the early pandemic period [14]. We can partially reproduce these findings. In our study, men reported higher initial contact levels compared to women, too. Both genders showed a comparable reduction behavior, but without precise information on the number of reduced contacts, we do not know the final contact level after 18 March 2020. In terms of our findings indicating that people with high educational and occupational status showed the most initial professional contacts and highest reduction rates, we found limited comparable sources. Some studies augmented, that people working in the less qualified occupational sectors, e.g. care, retail and restaurant workers, have the most personal contacts and though the highest infection rates, too. [39, 40]. Unfortunately, these studies lack information on the real contacts rates in the working fields. In addition to the described socioeconomic differences in contact behavior, some studies found that lower socioeconomic status groups showed less strict adherence to physical distancing and other infection control measures during the pandemic [5, 41] as well as less private contact reductions in lower educational groups through more crowded living conditions [4, 5], which might further explain the socioeconomic inequalities in infections. Further, educational differences in the perceptions of health risk due to COVID-19 are discussed. However, the state of research is ambiguous in that case. For Germany, different studies found higher [42] versus lower risk perception of COVID-19 [21] in lower educated groups using different data sources. Pre-pandemic studies have shown in many cases that education is strongly correlated with health literacy [43, 44]. This may also be of particular importance for the COVID-19 pandemic, as a large amount of frequently changing information especially at the beginning of the pandemic confronted everyone, but may have particularly challenged low educated groups.

Less previous evidence and explanations can be found for the association between occupational status and contact reductions. Available evidence focuses on differences between professions in infection rates [38, 45] but without information on individual occupational status. While educational differences in reducing contacts might arise from differences in risk perception, perceived dangerousness or adherence to government-introduced mitigation strategies [21, 46], occupational differences might be more linked to the structural conditions that enable a reduction and rely on specifics of the working sector [39, 47, 48]. Our results suggest that occupational status might be more strongly associated to the possibility to reduce professional contacts than educational status. Therefore, it appears to be probable that at least some workers in the lower occupational groups did not have the possibility to reduce their professional contacts regardless of their educational status. In addition to the differing amount of reduceable contacts in each occupational segment, it can be assumed that in the group with lower socioeconomic status there are more people in precarious employment and essential job conditions [49], who are less able to advocate for compliance with the distance regulations due to higher risks of job loss.

### Strengths and limitations

A strength of this study is its focus on socioeconomic determinants of the reduction of contacts in different settings during the first pandemic wave. Using individual level data, our study provides additional insights that contribute to a better understanding of the context in which different infection rates among socioeconomic groups have occurred. The data stems from population-representative samples of particularly affected municipalities during the first pandemic period in Germany. In terms of generalizability it should be noted that the municipalities studied are socioeconomically rather wealthy rural regions, and the results are not representative to Germany as a whole. In addition, due to the temporal dynamics in the pandemic, the results can be only partially generalized for later pandemic waves and lockdowns.

The definition of contacts in the CoMolo study could be of importance for our findings. Here, only contacts with longer face-to-face interaction and a physical distance less than 1.5 m were considered as contacts. These contacts might be more frequent in professions with close contacts than in other professions. One major limitation of this study is the lack of information on occupational sector. Linking data on occupational status and working sector would be a useful addition for future studies. Moreover, we interpreted the results according to the employable population in our sample but this population might be still heterogenous regarding social status. We therefore included different sociodemographic covariates in our multivariate analyses to control for this variability. However, there might be other relevant variables such as psychosocial factors that affect the contact behavior.

Another limitation is that we cannot say to which period the participants’ answer about the initial contact level refers to exactly. The persons may refer to the ultimate months before the survey, when COVID-19 incidence was already rising in Germany, or they may refer to the time before the pandemic. Furthermore, the results might be biased due to recall bias due to the retrospective design of the questions on contact reductions. Similarly, there is no information on the final contact level after 18 March 2020. We can only see the active step to have reduced private and professional contacts after that date, but not the intention or extent of reduction. However, these answers might be influenced by social desirability bias as well, as the study took part during an early stage of the pandemic where acceptance of mitigating measures was high. It would have been valuable to see whether socioeconomic differences in contact levels and contact reductions were associated with higher numbers of infections. However, the case numbers in our study population were too low for further stratification, so we statistically controlled for the occurrence of an own infection and personal contacts with infected persons. More differentiated mediation analyses would certainly be useful here and require longitudinal studies. Similar limitations apply to the variable working remotely, which is statistically adjusted here, but also could be seen as a mediating factor. Although the possibility to work from home is often discussed in the context of differences in infection rates by occupation [4], only one third of the common jobs in the German labor market is suitable for working from home, with large differences by region and degree of urbanization [50]. In this respect, a large proportion of the workforce remains tied to structural working conditions on site, and our findings indicate higher rates of not reducing professional contacts in lower occupational status groups, also when adjusting for working remotely, which seems to be more common with rising occupational status. Finally, we did not separately analyze the group of people that are not part of the regular labor market (e.g. students, training course participants, pensioners, people in parental leave). To avoid large numbers of missing values, we included them in the analyses. This might be important for the differing professional contact reduction gradients. Results on that group are not interpreted in the analyses by occupational status, whereas in the analyses by educational status these people are part of the three educational status groups.

## Conclusion

Our results point towards the necessity to adapt infection prevention and control strategies particularly at the workplace and to revise pandemic preparedness plans to consider the continued higher levels of contacts in certain educational and occupational status groups. Especially those in low occupational status groups were less able to reduce their professional contacts and might benefit from targeted support, such as the distribution of personal protective equipment for those who are not able to work remotely and reduce their physical contacts. Preventive measures that a) adequately explain the importance of contact restrictions with respect to varying living and working conditions and b) facilitate the implementation of these reductions especially in the occupational setting seem necessary to better protect structurally disadvantaged groups during epidemics.

## Supplementary Information

**Additional file 1.**

## Data Availability

The data used in this study are not publicly available at this time due to data protection regulations. However, access can be requested for scientific reasons. Requests should be submitted to the Research Data Centre at the Robert Koch Institute, Nordufer 20, 13,353 Berlin, Germany. E-mail: fdz@rki.de.
