# Supplementary material for: Socioeconomic differences in the reduction of face-to-face contacts in the first wave of the COVID-19 pandemic in Germany
Source: BMC Public Health. 2022 Dec 23;22:2419. doi: 10.1186/s12889-022-14811-4 (PMC9780616; doi:10.1186/s12889-022-14811-4)
Supplement: Supplementary file 1 — Additional file 1. [file 12889_2022_14811_MOESM1_ESM.docx]

**Additional File 1**

**
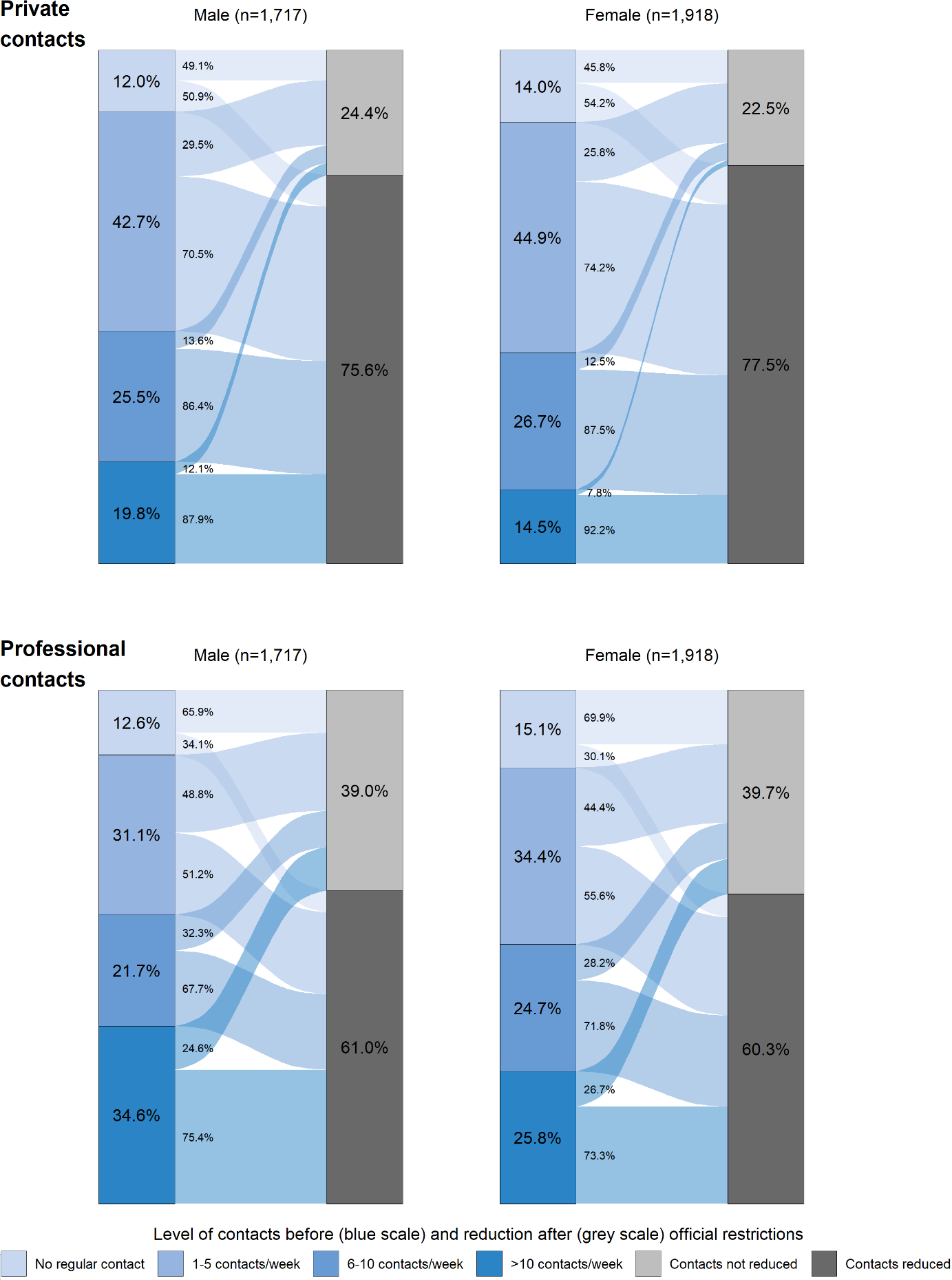
**

**Figure Appendix 1 Private and professional contact levels before and reduction after 18 March 2020 by gender.** n unweighted; % weighted according to data of the official population statistics regarding age, gender and education; Pearson’s χ²-test with Rao & Scott adjustment between gender and private/professional contact reduction: Private: p = 0.254; Professional: p = 0.734

**Tab. Appendix 1 Reduction of private and professional contacts after 18 March 2020 by educational and occupational status for municipality Kupferzell (n=1,381)**

|  | **Educational status** (ref. high) | | **Occupational status** (ref. high) | |
| --- | --- | --- | --- | --- |
| **Reduction of private contacts** | Low | Medium | Low | Medium |
| M1a: Confounder model* | 0.80 (0.65;0.99)  **p=0.036** | 0.95 (0.90;1.00)  p=0.068 |  |  |
| M2a: M1a + adjusting for occupational status | 0.81 (0.66;1.00)  p=0.053 | 0.96 (0.91;1.02)  p=0.212 |  |  |
| **Reduction of professional contacts** |  |  |  |  |
| M1b: Confounder model* | 0.77 (0.60;0.99)  **p=0.041** | 0.82 (0.75;0.89)  **p<0.001** | 0.61 (0.53;0.71)  **p<0.001** | 0.79 (0.73;0.85)  **p<0.001** |
| M2b: M1 + adjusting for occupational resp. educational status | 0.86 (0.66;1.12)  p=0.262 | 0.88 (0.81;0.97)  **p=0.007** | 0.65 (0.56;0.76)  **p<0.001** | 0.82 (0.76;0.89)  **p<0.001** |
| M3b: M2b + adjusting for working remotely | 0.93 (0.72;1.20)  p=0.578 | 0.92 (0.85;1.01)  p=0.077 | 0.70 (0.60;0.82)  **p<0.001** | 0.86 (0.80;0.93)  **p<0.001** |

*adjusting for age, gender, country of birth, household size, contact level before 18 March 2020, contact to Sars-cov-2 infected people, own infection lifetime; Significant p-values (p < 0.05) in bold

**Tab. Appendix 2 Reduction of private and professional contacts after 18 March 2020 by educational and occupational status for municipality Bad Feilnbach (n=1,275)**

|  | **Educational status** (ref. high) | | **Occupational status** (ref. high) | |
| --- | --- | --- | --- | --- |
| **Reduction of private contacts** | Low | Medium | Low | Medium |
| M1a: Confounder model* | 0.77 (0.63;0.95)  **p=0.015** | 0.91 (0.85;0.97)  **p=0.008** |  |  |
| M2a: M1a + adjusting for occupational status | 0.80 (0.65;1.00)  **p=0.046** | 0.94 (0.87;1.01)  p=0.109 |  |  |
| **Reduction of professional contacts** |  |  |  |  |
| M1b: Confounder model* | 0.76 (0.55;1.06)  p=0.109 | 0.79 (0.72;0.87)  **p<0.001** | 0.55 (0.45;0.67)  **p<0.001** | 0.79 (0.72;0.87)  **p<0.001** |
| M2b: M1 + adjusting for occupational resp. educational status | 0.89 (0.63;1.24)  p=0.485 | 0.90 (0.81;1.00)  p=0.056 | 0.58 (0.48;0.71)  **p<0.001** | 0.83 (0.74;0.91)  **p<0.001** |
| M3b: M2b + adjusting for working remotely | 0.95 (0.67;1.33)  p=0.748 | 0.91 (0.83;1.01)  p=0.088 | 0.67 (0.54;0.82)  **p<0.001** | 0.92 (0.83;1.02)  p=0.117 |

*adjusting for age, gender, country of birth, household size, contact level before 18 March 2020, contact to Sars-cov-2 infected people, own infection lifetime; Significant p-values (p < 0.05) in bold
